# Acceptance of Wife Beating and Its Association with Physical Violence towards Women in Nepal: A Cross-Sectional Study Using Couple’s Data

**DOI:** 10.1371/journal.pone.0095829

**Published:** 2014-04-21

**Authors:** Kayoko Yoshikawa, Tara M. Shakya, Krishna C. Poudel, Masamine Jimba

**Affiliations:** 1 Department of Community and Global Health, Graduate School of Medicine, The University of Tokyo, Tokyo, Japan; 2 Public Health Concern Trust, Kathmandu, Nepal; 3 Department of Public Health, School of Public Health and Health Sciences, University of Massachusetts-Amherst, Amherst, Massachusetts, United States of America; Tulane University, United States of America

## Abstract

**Background:**

Intimate partner violence (IPV) is a serious global public health issue. Acceptance of wife beating is known to be associated with IPV, but few studies have analysed the acceptance of wife beating from both women and men’s points of view. The objective of this study was to examine whether acceptance of wife beating among couples is associated with lifetime and past one-year physical IPV perpetration towards wives in Nepal.

**Methods:**

A cross-sectional study was conducted from August to September 2011, with 717 randomly selected couples with wives aged 18 to 49 years old from the Kirtipur municipality and Bhaktapur district of Nepal. Wives’ and husbands’ acceptance of wife beating was measured by six scale items, while physical IPV experience among wives was measured by seven physical assault scale items. To assess the association between acceptance of wife beating and physical IPV, multiple logistic regression analysis was used.

**Results:**

Nearly 30% of wives and husbands indicated that beating of wives is acceptable under certain circumstances. Statistically, no significant difference was detected between wives’ and husbands’ level of acceptance of wife beating. However, husbands’ acceptance of wife beating was positively associated with lifetime and past one-year perpetration of physical IPV, whereas wives’ acceptance of wife beating was neither associated with lifetime nor past one-year victimization of physical IPV. The positive association for husbands remained even after controlling for their partner’s factors.

**Conclusions:**

Acceptance of wife beating is an important risk factor, which must be considered to prevent perpetration of physical IPV towards wives in Nepal. Future studies should include men to better understand the structure and dynamics of IPV in Nepal, and prevention programs should also target men to change their attitudes or to identify which couples are at more risk of physical IPV occurring toward wives.

## Introduction

Intimate Partner Violence (IPV) is a serious health and social issue. The WHO multi-country study estimated that violence towards women occurs across many countries and cultures at varying magnitudes [1]. IPV has been implicated in many health problems among victimized women; such as physical injuries, headaches [2], [3], depressive symptoms [3], HIV/AIDS infection [2], [4], and adverse pregnancy outcomes [5], [6], [7], [8].

Numerous reports have reviewed the factors associated with IPV experience. Younger age increases the risk of either victimization or perpetration of current IPV [5], [9]. Having low educational attainment has been consistently found to increase the risk for being a victim or perpetrator of violence [5], [7], [10]. Though evidence sometimes points to the opposite relationship [5], higher socioeconomic status decreased risk of IPV in most studies [7], [10], [11]. Husbands who consume alcohol also have significantly higher risk of perpetrating IPV towards wives [2], [5], [7], [9], [11]. Exposure to violence during childhood is known to be another important risk factor. If a person witnessed his/her mother being abused by their father, or they themselves had been regularly abused by family members during childhood, the risk of IPV occurring in that individual’s future relationships increases significantly [2], [7], [10].

Wives who accept wife beating are more likely to experience IPV from husbands than wives who do not [7], [9]. Women’s positive attitudes to male dominance were the strongest predictor of physical violence among other risk factors in women who visited public health services in Iran [12]. Predictors of such attitudes among women have been widely examined. A study conducted in Bangladesh found that acceptance of wife beating is more prevalent among women with older age, with higher decision-making authority, and who had been exposed to physical violence during the previous 12 months [13]. Women whose fathers abused their mothers have also been found to have more supportive attitudes toward wife beating, and this has also been found to be the case among men [14], [15].

Despite the large volume of evidence from women, few studies have focused on the attitudes of husbands towards violence. The limited studies that have been conducted, however, suggest that men’s acceptance of wife beating is associated with increased risk of IPV perpetration against women [9]. A study from Bangladesh indicated that having some wife beating norms increases the risk of perpetration of lifetime and past-year physical IPV [16]. Such attitudes also increased the risk of perpetration of sexual IPV during past 10 years among men in South Africa [17]. However, the evidence focusing on men’s factors is still scarce, and no studies have been conducted thus far into the attitudes towards wife beating among men in Nepal.

Nepal is among the poorest and least developed countries in the world. Almost one-third of its population lives below the poverty line [18]. Women’s status is still low, as is reflected in their literacy rate [19], maternal mortality ratio [20], and Nepal’s rank in the Human Development Index [21]. Research on IPV against women has just recently started to receive attention in Nepal. Reported lifetime prevalence of physical IPV among ever-married women in Nepal was 23.3% in 2008 [22] and 23.1% in 2011 [23], while past one-year prevalence of physical IPV was 10% in 2011 [23]. About 23% of women and 21% of men believed that a husband was justified in beating his wife for at least one of the specified reasons, such as if she burns the food, if she argues with him, if she goes out without telling him, if she neglects the children, and if she refuses to have sexual intercourse with him [24]. Women with no education and women in the lower wealth quintile were more likely to accept wife beating [24]. Among men, those with younger age, lower education, and who were employed but not for cash were more likely to accept wife beating [24].

As these statistics imply, Nepalese culture has elements of a strong patriarchal society. In patriarchal societies fathers and husbands typically dominate the family and influence women’s lives. Women are expected to be subordinate to their husband and to devote themselves to domestic house chores and taking care of family members. These prevailing views and attitudes towards violence among Nepalese couples may differently influence the association between acceptance of wife beating and IPV in Nepal compared with other countries. A study conducted in rural Nepal revealed that acceptance of wife beating among married women was not associated with physical or sexual IPV victimization in the past 12 months [25], which is not in line with evidence from other countries.

In Nepal, factors that may predict husband’s perpetration of IPV, including acceptance of wife beating, have not yet been fully studied. Taking the patriarchal culture of Nepal into account, the potential association of husband’s acceptance of wife beating with physical IPV occurrence among Nepalese couples needs to be examined. Therefore, the purposes of the present study were to examine how acceptance of wife beating differs between husband and wife within couples, and to assess whether couples’ acceptance of wife beating is associated with physical IPV towards wives. This study is unique in that it uses couple data to examine the association of both wives’ and husbands’ factors with physical IPV simultaneously.

## Methods

### Setting

This study is a community-based, cross-sectional study conducted in the Kirtipur municipality and Bhaktapur district in the Kathmandu Valley, Nepal, during August and September 2011. The Kathmandu Valley consists of three districts: the Kathmandu District, Laltipur District, and Bhaktapur District. Kirtipur municipality is one of the municipalities contained within Kathmandu District, in which Kathmandu Metropolitan City (the capital of Nepal) also resides. Bhaktapur District is made up of Bhaktapur municipality, Madhyapur Thimi Municipality, and several village development communities. Based on the Census 2001, the population of Kirtipur was 40,835 and that of Bhaktapur and Madhyapur Thimi were 72,543 and 45,751, respectively. We selected these municipalities as the study areas because these areas are the home of the indigenous people called Newar, and therefore are expected to reflect the lives of people originally residing in the Valley.

### Study Population

The target population of this study was couples living in Kirtipur, Bhaktapur, and Madhyapur Thimi municipalities. We included couples if they had been married for at least one year preceding the interview and the age of the wife was between 18 to 49 years old. Couples were excluded if they were not cohabitating in the same house and if either or both couple had suffered from severe physical or mental illnesses. In the case of more than two couples in the same household, one couple was selected randomly.

### Sampling Methods

Sample size was calculated using Epi info 6. From previous studies, we assumed that the prevalence of wife beating being considered acceptable under certain situations among Nepalese women was 30% [24] and the prevalence of physical violence ever experienced among Nepalese women was 25% [22]. With 95% confidence interval (CI) and 80% power, the sample size required to detect 2.0 odds ratio for attitudes on IPV experience was 383. Considering expected design effect (1.8) of using cluster sampling, the sample size required for this study turned out to be 689 couples. In case of sampling non-eligible couples and missing information, we decided to approach 804 couples in total. This total sample size was equally divided into Kirtipur municipality and Bhaktapur district, and therefore 402 couples were approached in each district. From the analysis, 46 couples were dropped since they did not complete the interview due to ineligibility, absences or refusal. Further, 33 couples were dropped due to inconsistent matching of wife and husband, and 8 couples who had missing information on critical variables. Those variables were wives’ violence (n = 2), husband’s violence (n = 1), education (n = 4), and husband’s age (n = 1). Finally, 717 couples remained for analysis.

Two-stage cluster sampling methods were adopted to select participants. First, samples from Kirtipur municipality and Bhaktapur district (Bhaktapur and Madhyapur Thimi municipalities) were constructed as two separate domains. Each municipality has 9 to 35 wards. Wards are the smallest administrative units and the number of households contained in a ward widely varies. Using the election voter's list for 2005/2006 (Kirtipur) and the resident list for 2001 (Bhaktapur and Madhyapur Thimi) as sampling frames, six clusters (wards) were selected from each domain by probability proportional to size sampling. In Bhaktapur districts, four wards from Bhaktapur municipality and two wards from Madhyapur Thimi municipality were selected. Then, a fixed number of households (67 households each) were selected from each ward by random selection using a systematic sampling method.

### Questionnaires

Two questionnaires were prepared, one each for women and men, based on the WHO multi-country study for domestic violence against women. The WHO questionnaire maintained comparability to facilitate cross-cultural use and comparison [26] and therefore has been used worldwide [1], including in studies conducted in Nepal [22], [27]. In this study, the men’s questionnaire was constructed by converting the words “your husband” and “him” into “your wife” and “her”. The questionnaires were translated into Nepali and back translated into English by native Nepali speakers who were not familiar with the questionnaire. Pre-test was conducted with 30 couples to check the flow of questionnaires, and readability and acceptability of the contents of the questionnaires. The questionnaires were administered by face-to-face interview due to low literacy rate of Nepalese women. Finally, data were double entered into EpiData 3.1.

### Exposure to Physical Violence

The outcome variables in this study were lifetime as well as past one-year victimization or perpetration of physical IPV of wives and husbands. In the WHO questionnaire, violence was measured based on the tradition of the Conflict Tactic Scale 2 (CTS2) [28]. The CTS2 has been used for international comparisons around the world [5] since its approach is to ask about specific violent behaviors or acts and avoids emotional or cognitive appraisal of violence [29]. In the WHO questionnaire, seven physical assault scale items were used. Items asked to respondents were: “slapping”, “pushing, shoving or pulling hair”, “hitting”, “kicking, dragging, or beating”, “choking or burning”, or “threatening with weapon”. Respondents who answered ‘yes’ to any lifetime/past one-year physical assault items were coded as ‘1’ and those answered ‘no’ to all lifetime/past one-year physical assault items were coded as ‘0’. Prevalence obtained from both members of the couples was counted when at least one member of the couples reported a violent event, which is called the upper-bound estimate [30].

### Acceptance of Physical Violence

In this study, the independent variable was husband and wives’ acceptance of physical violence. Questions on the acceptability of physical violence included whether respondents accept wife beating if “wife does not complete household work”, “wife disobeys”, “wife refuses sex”, “wife doubts whether husband has other girlfriends”, “husband suspects that wife is unfaithful”, and “husband finds that wife is unfaithful.” Respondents giving the answer of “no” to all of the acceptability of physical violence items were coded as ‘0’ and regarded as “not accepting of violence”. Respondents giving an answer of “yes” to any items on the acceptability of physical violence were coded as ‘1’ and regarded as “accepting of violence”.

### Sociodemographic Characteristics

Factors that could confound the association were also obtained. They included age, education, caste, region, wife’s work, number of children, and household income. Ages of wives were classified as “less than 30”, “31–35”, “36–40” and “more than 40”, while that of husbands were “less than 33”, “34–39”, “40–45” and “more than 45”. Education of both wives and husbands was categorized into none, primary, secondary, and higher. Caste was classified as “Newar” or “others”. Others included Brahman/Chetri, Dalits, Janajati, and those with no answer. Wife’s work was asked in terms of whether the wife has jobs or not. Information about the number of children that was collected from wives was categorized into “0–1”, “2”, or “more than 3”. Household income was categorized into “<4,000 Rupees”, “4,000–30,000 Rupees”, “>30,000 Rupees”, and “don’t know/no answer”. As for witnessing father hitting mother and whether the respondents were beaten regularly by someone in the family during childhood, respondents who gave “no” to the question were coded as ‘0’ and ‘1’ if the respondents gave “yes”.

### Data Analysis

In this study the target population was couples, therefore the dyad was treated as the unit of analysis and hence the total sample size was 717. First, couple’s characteristics were described using means and percentage. Then the difference of acceptance of wife beating between wives and their husbands was compared with the chi-square test. Prevalence of physical IPV reported by couples was also compared using the chi-square test. Association between acceptance of wife beating and physical IPV was assessed by multiple logistic regressions. Three analytical models were fitted for IPV experience, which was assessed by combining the prevalence from wives and husbands. Model 1 explored wives’ factors and common factors (wife’s work, household income, number of children, and region) on physical IPV. Model 2 examined husbands’ factors and common factors. Model 3 added both wives’ and husbands’ factors into the model. Results were given as odds ratios (OR) and 95% CI with significance level set at 0.05.

### Ethical Issues

Ethical approval was granted from the Nepal Health Research Council and the Research Ethics Committee of the University of Tokyo.

All female and male interviewers received training, with particular focus on dealing with sensitive topics such as IPV. Interviewers were counsellors or social workers working at local NGOs (Kirtipur) or those who had medical backgrounds such as nurses or medical students (Bhaktapur and Madhyapur Thimi). Pairs of female and male interviewers were recommended to visit selected couples together for the first time, to identify the same households and couples. If the person selected was not available at that time, they made an appointment to return to conduct the interview at another time. When requesting participation, interviewers were guided to only refer to the study as a study on ‘health and life experiences of couples’, not ‘violence experience of couples’. They were told not to show the questionnaires to anyone, even before the questionnaire was answered.

Informed consent was obtained from all participants. For those who agreed to participate, their signature was obtained before conducting the survey. If participants were illiterate, a thumbprint was obtained instead. The respondent’s wishes about when and where to be interviewed were respected. The interviewers ensured that interviews would be conducted separately from their spouses and that no one else other than children less than 2 years old would be present. All participants were informed about confidentiality and the voluntary nature of participation. Participants were able to stop the interview at any time, skip any questions that they did not want to answer, or withdraw from the interview whenever they wanted to.

## Results

### Characteristics of Study Population

The mean age of wives was 35.6 (SD 7.1) years old and of husbands was 38.8 (SD 7.6) years old (Table 1). Their ages were categorized so that the numbers of participants in each category was balanced. Nearly 30% of women received no education (32.2%) and a further 30% had secondary education (28.7%), while nearly 80% of men had at least secondary education. As expected, most of the participants were Newar (wives 88.4%; husbands 89.8%) in these regions. Half of women did some work (52.7%) whereas almost all men engaged in some work (98.9%). Regarding exposure to violence during childhood, nearly 10% of participants reported that their mother was beaten by their father (wives 9.2%; husbands 10.6%) or having suffered regular abuse from family members in their childhood (wives 5.4%; husbands 12.8%).

**Table 1:** Sociodemographic characteristic of Nepalese couples.

	Wives (N = 717)		Husbands (N = 717)	
	N	%	N	%
Current age				
≦30	189	26.4	–	–
31–35	159	22.2	–	–
36–40	153	21.3	–	–
≧41	216	30.1	–	–
Mean (SD)	35.6 (7.1)		–	–
Current age				
≦33	–	–	198	27.6
34–39	–	–	187	26.1
40–45	–	–	196	27.3
≧46	–	–	136	19
Mean (SD)	–	–	38.8 (7.6)	
Education				
None	231	32.2	37	5.2
Primary	153	21.3	109	15.2
Secondary	206	28.7	364	50.8
Higher	127	17.7	207	28.9
Caste				
Newar	634	88.4	644	89.8
Others	83	11.6	73	10.2
Work				
Do not work	339	47.3	8	1.1
Do work	378	52.7	709	98.9
Mother beaten by father				
No	624	87.0	542	75.6
Yes	66	9.2	76	10.6
Don’t know/others	27	3.8	99	13.8
Regularly beaten as a child				
No	651	90.8	594	82.9
Yes	39	5.4	92	12.8
Don’t know/others	27	3.8	31	4.3
Household income				
4,000–30,000 Rupees	598	83.4	–	–
<4,000 Rupees	23	3.2	–	–
>30,000 Rupees	54	7.5	–	–
Don’t know/no answer	42	5.9	–	–
Region				
Kirtipur	366	51.1	–	–
Bhaktapur	351	49.0	–	–
Number of children				
0–1	199	27.8	–	–
2	322	44.9	–	–
More than 3	196	27.3	–	–

Couple variables are described in the wives’ column. Most couples had a household income of 4,000–30,000 rupees (83.4%), with few reporting less than 4,000 rupees (3.2%) or more than 30,000 rupees (7.5%). After selecting participants to be included in the analysis, 51% of couples were from Kirtipur district and 49% were from Bhaktapur district. Nearly 28% of couples had 0–1 children, and a further 28% had more than 3 children. The majority of couples had two children (44.9%).

### Comparison of Acceptance of Wife Beating

Acceptance of wife beating among wives and their husbands was compared using the chi-square test (Table 2). For each circumstance for the acceptability of violence asked about, more than 80% of couples answered that both of them do not accept wife beating, with the exception of when the husband found that his wife has been unfaithful (56.5%). Overall, about half of couples answered they do not accept wife beating under any of the circumstances presented in the questionnaire. Nearly half of the couples, however, answered that either or both of them accept the beating of wives under certain circumstances given in the questionnaires. When we compared the answer from wives with that from husbands, there were no significant differences between their responses. For overall acceptance of wife beating, the difference between wives and husbands was also not significant (p = 0.103).

**Table 2:** Comparison of acceptance of wife beating among Nepalese couples.

		Couples (N = 717)		
		N	%	P-value
She does not complete her household work				0.506
Wife not accept ×Husband not accept		643	89.7	
Wife not accept ×Husband accept		28	3.9	
Wife accept ×Husband not accept		45	6.3	
Wife accept ×Husband accept		1	0.1	
She disobeys him				0.629
Wife not accept ×Husband not accept		603	84.1	
Wife not accept ×Husband accept		51	7.1	
Wife accept ×Husband not accept		57	7.9	
Wife accept ×Husband accept		6	0.8	
She refuses to have sexual relations with him				0.314
Wife not accept ×Husband not accept		648	90.4	
Wife not accept ×Husband accept		26	3.6	
Wife accept ×Husband not accept		40	5.6	
Wife accept ×Husband accept		3	0.4	
She asks him whether he has other girlfriends				0.275
Wife not accept ×Husband not accept		630	87.9	
Wife not accept ×Husband accept		39	5.4	
Wife accept ×Husband not accept		47	6.6	
Wife accept ×Husband accept		1	0.1	
He suspects that she is unfaithful				0.753
Wife not accept ×Husband not accept		584	81.5	
Wife not accept ×Husband accept		63	8.8	
Wife accept ×Husband not accept		64	8.9	
Wife accept ×Husband accept		6	0.8	
He found that she has been unfaithful				0.659
Wife not accept ×Husband not accept		405	56.5	
Wife not accept ×Husband accept		122	17.0	
Wife accept ×Husband not accept		143	19.9	
Wife accept ×Husband accept		47	6.6	
Total acceptance of wife beating				0.103
Wife not accept ×Husband not accept		371	51.7	
Wife not accept ×Husband accept		135	18.8	
Wife accept ×Husband not accept		142	19.8	
Wife accept ×Husband accept		69	9.6	

### Comparison of Reports of Physical IPV

Physical IPV towards wives as reported by wives and their husbands was compared by chi-square test (Table 3). Most couples reported no physical IPV towards wives in their lifetime (79.4%; p<0.001). On the other hand, only 4.2% of couples agreed on lifetime occurrence of physical IPV. About 16% of couples showed disagreement with each other’s response. With regard to past one-year physical IPV towards wives, about 13% of couples disagreed with each other’s response. Similar to lifetime physical IPV, most couples answered there was no IPV in the past year, and this was statistically significant (p<0.001).

**Table 3:** Comparison of prevalence of lifetime and past one-year physical IPV among Nepalese couples.

	Couples (N = 717)		
	N	%	P-value
Lifetime physical IPV			<0.001
Wife NO ×Husband NO	569	79.4	
Wife NO ×Husband YES	67	9.3	
Wife YES×Husband NO	51	7.1	
Wife YES×Husband YES	30	4.2	
Past 1 year physical IPV			<0.001
Wife NO ×Husband NO	607	84.7	
Wife NO ×Husband YES	56	7.8	
Wife YES×Husband NO	38	5.3	
Wife YES×Husband YES	16	2.2	

### Multivariate Analysis of the Association between Acceptance of Wife Beating and Physical IPV

The association between acceptance of wife beating and lifetime physical IPV towards wives was assessed by multiple logistic regression (Table 4). Husbands’ acceptance of wife beating was positively associated with lifetime perpetration of physical IPV in all models (Model 2 Adjusted OR (AOR): 2.81, 95% CI: 1.57–5.05; Model 3 AOR: 2.58, 95% CI:1.36–4.91). For wives, their acceptance of wife beating was not associated with lifetime victimization due to physical IPV from their husband (AOR: 1.38, 95% CI: 0.74–2.55), even after controlling for husbands’ factors (AOR: 1.36, 95% CI: 0.76–2.43). Among confounding factors, only wives’ education was significantly associated with physical IPV; the higher the education, the lower the risk of IPV. However, this association was lost when we controlled husbands’ factors. Among husbands, having a mother who had been abused by a father positively increased the risk of physical IPV perpetration towards wives (Model 2 AOR: 2.13, 95% CI:1.20–3.76; Model 3 AOR: 1.92, 95% CI: 1.03–3.57). Likewise, husbands who had been regularly beaten as a child were more likely to perpetrate physical IPV (Model 2 AOR: 3.50, 95% CI: 2.11–5.80; Model 3 AOR: 3.25, 95% CI: 1.81–5.81).

**Table 4:** Association between acceptance of wife beating among couples and lifetime physical IPV towards wives.

		Model 1^a^	Model 2^b^	Model 3^c^
		AOR (95%CI)	AOR (95%CI)	AOR (95%CI)
**Wives’ factors**				
Acceptance of wife beating				
Not accepting		1.00	–	1.00
Accepting		1.38 (0.74–2.55)	–	1.36 (0.76–2.43)
Education				
None		1.00	–	1.00
Primary		0.91 (0.57–1.46)	–	1.01 (0.62–1.64)
Secondary		0.46 (0.21–0.98)*	–	0.64 (0.30–1.35)
Higher		0.39 (0.18–0.83)*	–	0.55 (0.25–1.23)
Mother beaten by father				
No		1.00	–	1.00
Yes		1.58 (0.99–2.50)	–	1.62 (0.99–2.68)
Don’t know/others		1.35 (0.67–2.72)	–	1.25 (0.65–2.42)
Regularly beaten as a child				
No		1.00	–	1.00
Yes		2.30 (0.96–5.51)	–	2.30 (0.96–5.51)
Don’t know/others		0.83 (0.35–1.98)	–	0.76 (0.32–1.80)
**Husbands’ factors**				
Acceptance of wife beating				
	Not accepting	–	1.00	1.00
	Accepting	–	2.81 (1.57–5.05)**	2.58 (1.36–4.91)**
Education				
None		–	1.00	1.00
Primary		–	0.93 (0.53–1.62)	0.90 (0.54–1.49)
Secondary		–	0.64 (0.34–1.22)	0.64 (0.34–1.22)
Higher		–	0.49 (0.23–1.05)	0.56 (0.26–1.23)
Mother beaten by father				
No		–	1.00	1.00
Yes		–	2.13 (1.20–3.76)**	1.92 (1.03–3.57)*
Don’t know/others		–	0.99 (0.47–2.08)	0.92 (0.42–2.02)
Regularly beaten as a child				
No		–	1.00	1.00
Yes		–	3.50 (2.11–5.80)***	3.25 (1.81–5.81)***
Don’t know/others		–	1.26 (0.45–3.48)	1.11 (0.32–3.84)

*p<0.05; **p<0.01; ***p<0.001. Notes: Model 1 explored wives’ factors and common factors (wife’s work, household income, number of children, and region) on physical violence. Model 2 examined husbands’ factors and common factors. Model 3 added both wives’ and husbands’ factors into the model.

a Controlled for wife’s age and caste, and common factors.

b Controlled for husband’s age and caste, and common factors.

c Controlled for wife’s and husband’s age and caste, and common factors.

The association of acceptance of wife beating with past one-year physical IPV was also investigated using multiple logistic regression (Table 5). As in the case of lifetime experience of IPV, wives’ report of acceptance of wife beating was not significantly associated with victimisation from physical IPV (Model 1 AOR: 1.65, 95% CI: 0.92–2.96; Model 3 AOR: 1.64, 95% CI: 0.93–2.91). On the other hand, husbands who accepted wife beating were more likely to perpetrate physical IPV towards wives (Model 2 AOR: 2.99, 95% CI: 1.60–5.58; Model 3 AOR: 2.78, 95% CI: 1.41–5.51). Wives’ higher educational status was a protective factor of IPV in model 1, but the association was not observed when husbands’ factors were included. On the contrary, husbands’ educational attainment was associated with lower risk of IPV perpetration in the past year in both model 2 and model 3. Moreover, husbands whose mother had been beaten by father were more likely to have perpetrated physical IPV in the past year (Model 2 AOR: 2.32, 95% CI: 1.36–3.94; Model 3 AOR: 2.27, 95% CI: 1.17–4.49). Having been regularly beaten during childhood was a significant risk factor for victimization/perpetration of physical IPV in the past year in both wives (Model 1 AOR: 4.40, 95% CI: 1.76–10.98; Model 3 AOR: 4.82, 95% CI: 1.97–11.80) and husbands (Model 2 AOR: 3.75, 95% CI: 2.03–6.93; Model 3 AOR: 23.15, 95% CI: 1.41–7.05), respectively.

**Table 5:** Association between acceptance of wife beating among couples and past 1-year physical IPV towards wives.

		Model 1^a^	Model 2^b^	Model 3^c^
		AOR (95%CI)	AOR (95%CI)	AOR (95%CI)
**Wives’ factors**				
Acceptance of wife beating				
	Not accepting	1.00	–	1.00
	Accepting	1.65 (0.92–2.96)	–	1.64 (0.93–2.91)
Education				
None		1.00	–	1.00
Primary		0.85 (0.58–1.26)	–	0.97 (0.66–1.43)
Secondary		0.41 (0.18–0.91)*	–	0.59 (0.26–1.38)
Higher		0.38 (0.18–0.79)**	–	0.53 (0.21–1.32)
Mother beaten by father				
No		1.00	–	1.00
Yes		1.13 (0.57–2.25)	–	1.02 (0.47–2.25)
Don’t know/others		1.61 (0.76–3.42)	–	1.54 (0.72–3.29)
Regularly beaten as a child				
No		1.00	–	1.00
Yes		4.40 (1.76–10.98)**	–	4.82 (1.97–11.80)**
Don’t know/others		1.35 (0.61–2.98)	–	1.32 (0.62–2.82)
**Husbands’ factors**				
Acceptance of wife beating				
Not accepting		–	1.00	1.00
Accepting		–	2.99 (1.60–5.58)**	2.78 (1.41–5.51)**
Education				
None		–	1.00	1.00
Primary		–	0.48 (0.23–1.03)	0.50 (0.23–1.08)
Secondary		–	0.37 (0.20–0.68)**	0.38 (0.19–0.74)**
Higher		–	0.29 (0.13–0.68)**	0.35 (0.13–0.93)*
Mother beaten by father				
No		–	1.00	1.00
Yes		–	2.32 (1.36–3.94)**	2.29 (1.17–4.49)*
Don’t know/others		–	0.74 (0.43–1.28)	0.69 (0.38–1.25)
Regularly beaten as a child				
No		–	1.00	1.00
Yes		–	3.75 (2.03–6.93)***	3.15 (1.41–7.05)**
Don’t know/others		–	2.33 (1.04–5.21)*	2.05 (0.62–6.77)

*p<0.05; **p<0.01; ***p<0.001. Notes: Model 1 explored wives’ factors and common factors (wife’s work, household income, number of children, and region) on physical violence. Model 2 examined husbands’ factors and common factors. Model 3 added both wives’ and husbands’ factors into the model.

a Controlled for wife’s age and caste, and common factors.

b Controlled for husband’s age and caste, and common factors.

c Controlled for wife’s and husband’s age and caste, and common factors.

## Discussion

In this study, the level of acceptance of wife beating between wives and their husbands was not significantly different. However, the husbands’ acceptance of wife beating was positively associated with lifetime and past one-year physical IPV towards wives, whereas wives’ acceptance of wife beating was not. The positive association remained even after controlling for wives’ factors.

Associations between husbands’ acceptance and perpetration of wife beating were also reported by the study from Bangladesh. The study explored risk factors of IPV and found that husbands’ acceptance of wife beating was one of the significant risk factors for lifetime and past one-year physical and sexual IPV in Bangladesh [16]. Bangladesh and Nepal have similar cultural features in common: a high proportion of positive attitudes towards violence [13] and a patriarchal society [31]. Such societies expects that men are dominant while women are dependent on their husband [32]. In Nepal, the discrepancy between men and women is also indicated in the literacy rate; women are less educated than men and have lower decision-making power [23], and sons are preferred to daughters [33], [34]. When men in a patriarchal society feel gender role stress or defensive masculinity, they may be prompted to reassert their dominance over women to cope with gender role conflict, from which IPV may originate [35], [36]. It is also possible that those husbands with positive attitudes may have a lower level of shame or hesitation to report IPV perpetration than those without. Hence it is likely that husbands who had positive attitudes towards violence were positively associated with IPV perpetration in these studies.

Our study also demonstrated that there was no significant association between acceptance of wife beating among women in relation to IPV occurring against wives. This is in accordance with earlier studies conducted in rural Nepal [25], [37]. Women’s acceptance of wife beating did not predict violence from husbands, though there was no significant difference in acceptance of wife beating between couples. This result suggests that rather than women’s attitudes towards violence, men’s attitudes are a more important focus for preventing IPV against wives, especially in a patriarchal society. Prevention programs should be required to include both husbands and wives, with particular focus on husbands who have positive attitudes towards wife beating.

The gender difference in the association brought an additional implication. In the field of IPV, most research tends to use the data from one gender, most often female. This is because women are often focused on their role as victims and many researchers are interested in the factors associated with being a victim, as well as the health consequences of violence. Therefore, female partners often provide information not only on their own characteristics but also on their partner’s sociodemographic characteristics, such as age and education, on their behalf. However, there are other important risk factors, such as acceptance of wife beating, for which female partners cannot provide information, as they are subjective measures and estimation by female partners would increase the risk of biased responses. Obtaining data from both couples is therefore critical when exploring such subjective factors.

A significant discrepancy was also detected between prevalence reported by couples. Husbands reported more IPV perpetration than wives’ report of IPV victimization.

As many researchers have postulated, the reasons for such discrepancies may be attributed to underreporting, biased recall, and memory and cognitive abilities of couples [30], [38], [39]. There are also other possible explanations for these discrepancies. Data collection methods, including interviewer errors and measurement errors, also affect the disagreement level. Because violence is a sensitive and private issue, and as for some couples it may be a daily event, there may be reluctance or hesitance to reveal the event. Efforts in collection methods may not be able to overcome these barriers, and therefore, prevalence reported from one-partner will be likely subject to some systematic errors. Hence, if the aim is to obtain the ‘true’ estimate of IPV prevalence, it is highly recommended that data is collected from both partners.

The present study has several limitations. Because there is no gold standard to estimate ‘true’ prevalence of IPV occurred among couples, we used upper-bound estimate of IPV prevalence in this study [30]. As such, there are possibilities of over-reporting by either member of couples. However, it is assumed that over-reporting would be less likely to occur than underreporting, unless under law enforcement. Under-reporting of one partner can be covered by the other partner in the upper-bound estimate. Thus we assumed that the upper-bound estimate would be more suitable for estimating the true prevalence of IPV in this study.

Another limitation is the cross-sectional nature of data. This limitation precluded us from making causal inferences. It is possible that the reverse relationship (i.e. IPV experience accelerates/decelerates acceptance of wife beating) could occur since acceptance of wife beating might be influenced by IPV experience in daily life, just as in experience of violence during childhood increasing risk of future violence. However, the same results occurring for the last one-year supports the likelihood of the proposed relationships in this study being accurate.

The third limitation relates to collecting data from couples at the same time. We do not know what impact the knowledge that both partners were answering the questionnaire may have had in responding to the interview. The couples might suspect that their partner may be able to guess what they were asked based on the questions their partner was asked. Despite interviewers’ efforts to conceal the contents of both partners’ questionnaire and to give assurance of confidentiality, it is possible that couples might have felt insecure and thus not shared their experiences with the interviewers.

Furthermore, collecting data from two communities, and avoiding data collection from Kathmandu Metropolitan City, limited the generalizability of the results to other couples residing outside the two communities.

Despite these limitations, the present study provides initial evidence to show the role played by acceptance of wife beating among Nepalese couples and its association with physical IPV towards wives. IPV is still a relatively new field of study in Nepal, and thus the present study added valuable information to the growing knowledge about IPV in relation to this country. Using couple data has methodological advantages in addressing the differences in impact of acceptance of wife beating in predicting physical IPV between couples. Future research should continue to identify causal inference between acceptance of wife beating and IPV in Nepal.

## Conclusions

This study described husbands’ attitudes towards wife beating in Nepal, and found that this was an important risk factor for lifetime and past one-year physical violence towards wives, rather than wives’ attitudes towards wife beating. Moreover, the study highlights the issues of obtaining ‘true’ estimates of IPV prevalence from couples. This is one of only a few studies that have examined men’s’ factors in relation to IPV in South Asia. The findings of this study highlight the importance of examining men’s factors, in addition to women’s factors, to understand the dynamics of IPV. Future research should continue to obtain couple’s risk factors and its association with IPV when investigating this issue in Nepal.

Changing attitudes towards violence is not an easy task. This study suggests future prevention program should attempt to change social norms at an earlier stage of life by addressing the cause of such attitudes and also by educating men, together with women. Screening attitudes in health facilities or in communities would also help to identify men and wives who are at risk of physical IPV occurring against the wife.
